# In vivo Ca^2+^ dynamics induced by Ca^2+^ injection in individual rat skeletal muscle fibers

**DOI:** 10.14814/phy2.13180

**Published:** 2017-03-14

**Authors:** Mario Wakizaka, Hiroaki Eshima, Yoshinori Tanaka, Hideki Shirakawa, David C. Poole, Yutaka Kano

**Affiliations:** ^1^ Department of Engineering Science, Bioscience and Technology ProgramUniversity of Electro‐CommunicationsChofuTokyoJapan; ^2^ Department of Metabolism and EndocrinologyJuntendo University Graduate School of MedicineHongoTokyoJapan; ^3^ Departments of Anatomy & Physiology and KinesiologyKansas State UniversityManhattanKansas

**Keywords:** Ca^2+^‐induced Ca^2+^ release, ryanodine receptor, sarcoplasmic reticulum, store overload‐induced Ca^2+^ release

## Abstract

In contrast to cardiomyocytes, store overload‐induced calcium ion (Ca^2+^) release (SOICR) is not considered to constitute a primary Ca^2+^ releasing system from the sarcoplasmic reticulum (SR) in skeletal muscle myocytes. In the latter, voltage‐induced Ca^2+^ release (VICR) is regarded as the dominant mechanism facilitating contractions. Any role of the SOICR in the regulation of cytoplasmic Ca^2+^ concentration ([Ca^2+^]_i_) and its dynamics in skeletal muscle in vivo remains poorly understood. By means of in vivo single fiber Ca^2+^ microinjections combined with bioimaging techniques, we tested the hypothesis that the [Ca^2+^]_i_ dynamics following Ca^2+^ injection would be amplified and fiber contraction facilitated by SOICR. The circulation‐intact spinotrapezius muscle of adult male Wistar rats (*n *=* *34) was exteriorized and loaded with Fura‐2 AM to monitor [Ca^2+^]_i_ dynamics. Groups of rats underwent the following treatments: (1) 0.02, 0.2, and 2.0 mmol/L Ca^2+^ injections, (2) 2.0 mmol/L Ca^2+^ with inhibition of ryanodine receptors (RyR) by dantrolene sodium (DAN), and (3) 2.0 mmol/L Ca^2+^ with inhibition of SR Ca^2+^ ATPase (SERCA) by cyclopiazonic acid (CPA). A quantity of 0.02 mmol/L Ca^2+^ injection yielded no detectable response, whereas peak evoked [Ca^2+^]_i_ increased 9.9 ± 1.8% above baseline for 0.2 mmol/L and 23.8 ± 4.3% (*P* < 0.05) for 2.0 mmol/L Ca^2+^ injections. The peak [Ca^2+^]_i_ in response to 2.0 mmol/L Ca^2+^ injection was largely abolished by DAN and CPA (−85.8%, −71.0%, respectively, both *P* < 0.05 vs. unblocked) supporting dependence of the [Ca^2+^]_i_ dynamics on Ca^2+^ released by SOICR rather than injected Ca^2+^ itself. Thus, this investigation demonstrates the presence of a robust SR‐evoked SOICR operant in skeletal muscle in vivo.

## Introduction

Precise control of cytoplasmic calcium ion concentration ([Ca^2+^]_i_) is crucial for regulation of myocyte function and the excitation–contraction (E–C) coupling process in particular. In skeletal muscle myocytes, contraction and relaxation are evoked by [Ca^2+^]_i_ increase and decrease, respectively. The primary Ca^2+^ releasing systems from the sarcoplasmic reticulum (SR) are action potential‐induced calcium release (voltage‐induced Ca^2+^ release; VICR) (Schneider 1994; Hernandez‐Ochoa et al. 2015), Ca^2+^‐induced Ca^2+^ release (CICR) (Endo 2009) and store overload‐induced Ca^2+^ release (SOICR) (Kong et al. 2007; Prakriya and Lewis 2015).

The ryanodine receptor (RyR) is a Ca^2+^ releasing channel on the SR membrane. In mammals, three RyR isoforms exist (RyR1, RyR2 and RyR3) with RyR1 being dominantly expressed in skeletal (Nakai et al. 1990; Otsu et al. 1990; Zorzato et al. 1990) as opposed to RyR2 in cardiac muscle (Nakai et al. 1990; Otsu et al. 1990; Zorzato et al. 1990). RyR3, discovered initially in the brain, also is present in the skeletal muscle (Giannini et al. 1995). However, RyR3 expression disappears in hind limb muscles of adult animals (Flucher et al. 1999). RyR is opened by interaction with the dihydropyridine receptor (DHPR), which is the voltage‐dependent Ca^2+^ channel of the transverse tubule membrane (Lanner et al. 2010). Myocardial RyR2 causes Ca^2+^ release by binding with the Ca^2+^ which flows into the cytosol across the sarcolemma (Fabiato 1992; Maack and O'Rourke 2007). In marked contrast, skeletal muscle RyR1 is opened by the structural change in the RyR1‐DHPR conjugate (Beam and Bannister 2010). Because this opening occurs in the absence of Ca^2+^ influx from the extracellular space, unlike that for cardiomyocytes, it is thought that a rise in [Ca^2+^]_i_ is not necessary for RyR1‐mediated SR Ca^2+^ release. However, not all SR RyR1 is in contact with DHPR. Thus, the presence of non‐DHPR‐associated RyR1 in skeletal muscle myocytes raises the possibility that these particular RyR1′s may release Ca^2+^ in a process instigated by Ca^2+^ that is released from the conjugated DHPR‐RyR1 (Klein and Schneider 2006; Pouvreau et al. 2007; Van Petegem 2012).

Although SOICR is well known to operate via RyR2 in cardiomyocytes, it has never been observed for RyR1‐bearing skeletal muscle myocytes even under conditions propagating Ca^2+^release via the SR (Zhou et al. 2004; Figueroa et al. 2012). Interestingly, however, the Ca^2+^ sensing gate on the RyR for SOICR has been identified as a common gating structure for all RyR isoforms (Chen et al. 2014) and Ca^2+^ release via SOICR does occur in skeletal muscle myocytes similar to cardiac myocytes expressing RyR2 (Cully et al. 2014). In addition, reduction of Ca^2+^ entry across the sarcolemma reduces SR Ca^2+^ accumulation and induces muscle fatigue (Stiber et al. 2008). Collectively, these findings suggest that Ca^2+^ release by SOICR might play an important role in skeletal muscle in vivo.

Because RyR regulation is extremely sensitive to the cytosolic and luminal environments, and changes thereof (Laver et al. 2000; Fill and Coppell 2002; Capes et al. 2011), direct evidence for the presence of SOICR must be obtained under in vivo conditions especially as regards pH, temperature, reactive O_2_ species, and O_2_ partial pressure. The present investigation was designed to resolve the presence and putative importance of SOICR in an in vivo skeletal muscle using Ca^2+^ solutions injected directly into the myocyte cytosol which bypasses the VICR process entirely. We hypothesized that the [Ca^2+^]_i_ change in response to the Ca^2+^ solution injection would be amplified by SOICR, raising [Ca^2+^]_i_ substantially and causing muscle contraction. Furthermore, it was hypothesized that this amplification would be either abolished or reduced when Ca^2+^ release from the SR is inhibited, either directly via the blockade of RyR by the hydantoin derivative dantrolene (DAN), or indirectly via the depletion of Ca^2+^ from the SR by inhibiting SERCA with cyclopiazonic acid (CPA).

## Methods

### Animals

Male Wistar rats (*n *=* *34, 10–14 weeks of age; Japan SLC, Shizuoka, Japan) were used in this study. Rats were maintained on a 12:12‐h light/dark cycle and received food and water ad libitum. All experiments were conducted under the guidelines established by the Physiological Society of Japan and were approved by the University of Electro‐Communications Institutional Animal Care and Use Committee. The rats were anesthetized using pentobarbital sodium (60 mg/kg i.p.), and supplemental doses of anesthesia were administered as needed. At the end of the experimental protocols, animals were killed by pentobarbital sodium overdose.

### Muscle preparation

All experimental techniques, including the spinotrapezius muscle preparation, were performed, as described previously (Sonobe et al. 2008; Eshima et al. 2013). Briefly, the right spinotrapezius muscle was gently exteriorized with minimal blood loss and tissue/microcirculatory damage, and attached to a wire horseshoe around the caudal periphery by six equidistant sutures placed around the muscle perimeter. The exposed muscle tissue was kept moist by superfusing with warmed Krebs–Henseleit buffer (KHB; 132 mmol/L NaCl, 4.7 mmol/L KCl, 21.8 mmol/L NaHCO_3_, 2.0 mmol/L MgSO_4_, and 2.0 mmol/L CaCl_2_) equilibrated with 95% N_2_‐5% CO_2_ and adjusted to pH 7.4, at 37°C. The fluorescent Ca^2+^ indicator Fura‐2 AM (5 mmol/L; Dojindo Laboratories, Kumamoto, Japan) was dissolved in dimethyl sulfoxide (DMSO) and Pluronic F‐127 and dispersed into KHB solution at a final concentration of 40 *μ*mol/L. The muscles were incubated in Fura‐2 AM/KHB solution for 60 min on a 37°C hot plate (Kitazato Supply, Shizuoka, Japan). After incubation, muscles were rinsed with dye‐free KHB solution to remove nonloaded Fura‐2 AM.

### In vivo image analysis

The spinotrapezius muscles loaded with Fura‐2 AM were mounted on the 37°C glass hot plate and observed by fluorescence microscopy using a 10 × objective lens (0.30 numerical aperture; Nikon, Tokyo, Japan). After ensuring that the spinotrapezius muscle was not grossly damaged and supported robust capillary blood flow, a sampling area (∼880 × 663 *μ*m) was selected, and bright‐field images were captured. Thereafter, 340 and 380 nm wavelength excitation light was delivered using a Xenon lamp equipped with appropriate fluorescent filters, and pairs of fluorescence images were captured through the 510 nm emission wavelength filter for ratiometry (Roe et al. 1990). The fluorescence intensity of serial ratio images was normalized to the starting point (i.e., precondition, R0) of each experiment (R/R0).

### Experimental Protocols

#### In vivo injection of Ca^2+^ solution

Capillary micropipettes were generated with a tip of 5 *μ*m in diameter, which was achieved by custom grinding and inserted into the selected single muscle fiber using a micromanipulator precision‐controlled advancer (MMO‐220A; Narishige, Tokyo, Japan). Subsequently, using a microinjector (IM300; Narishige, Tokyo, Japan), a single muscle fiber was microinjected with 0.02, 0.2, and 2.0 mmol/L Ca^2+^ solution by microinjection at 35 psi (24,000 Pa) for 1 sec (∼0.78 × 10^−3^ *μ*L) (Tanaka et al. 2016). We used a piezoassisted micromanipulation system for perforation of the cell membrane (PiezoXpert; Eppendorf, Hamburg, Germany). The injection solution was adjusted using a mixture of Ca^2+^‐free solution (KCl: 145 mmol/L, NaCl: 3.5 mmol/L, MgCl_2_: 10 mmol/L,NaOH: 6.5 mmol/L, HEPES: 10 mmol/L) and the Ca^2+^ solution (CaCl_2_・2H_2_O: 10 mmol/L). Criteria for successful injection included an unchanged [Ca^2+^]_i_ in adjacent muscle fibers. Fluorescence images were captured by a high‐sensitivity CCD (charge‐coupled device) digital camera (ORCA‐Flash2.8; Hamamatsu Photonics, Hamamatsu, Japan) using image‐capture software (NIS‐Elements Advanced Research; Nikon, Tokyo, Japan) for 60 sec after injection. The scan requirements were set at 300–800 msec/image. We confirmed that insertion of the capillary micropipette itself and injection of Ca^2+^‐free solution did not induce any [Ca^2+^]_i_ change. The specific region of interest (ROI, width: 20 *μ*m, height: 80% of muscle fiber diameter) for the Ca^2+^ measurement was set at a distance of 40 *μ*m from the injection point. All experiments were performed with the rat's body temperature maintained at 37–38°C with the exteriorized muscles mounted on the 37°C glass hotplate.

#### Pharmacologic block of the SR function

Pharmacological agents DAN and CPA were purchased from Sigma‐Aldrich (St. Louis, MO). DAN inhibits single ryanodine receptor (RyR) Ca^2+^ release channels (Kobayashi et al. 2005; Oo et al. 2015). CPA is a highly specific and potent SERCA inhibitor (Seidler et al. 1989). Each inhibitor was dissolved in DMSO and diluted with KHB (DAN: 100 and 500 *μ*mol/L, CPA: 10, 50, and 100 *μ*mol/L) and the pharmacological action was confirmed by constructing dose–response curves. Based upon these studies, following Fura‐2 incubation and rinse, DAN (100 *μ*mol/L) or CPA (100 *μ*mol/L) was applied for 5 min before the start of experiments. Subsequently, a single muscle fiber was microinjected with either of the 0.02, 0.2, and 2.0 mmol/L Ca^2+^ solution.

#### In vivo injection of caffeine solution

This protocol was the same as for the Ca^2+^ injection. The caffeine solution (100 *μ*mol/L) was injected into each single muscle fiber both with and without DAN conditions.

### Evaluation of the muscle fiber length

The muscle fiber shortening (i.e., contraction rate) was calculated frame‐by‐frame using autologous fluorescent landmarks, located close to the injection point and thus ~40 *μ*m from the ROI. The resolution was 0.73 *μ*m per pixel.

### Statistical analysis

All experimental data are expressed as means ± SE. All statistical analyses were performed in Prism version 6.0 (GraphPad Software, San Diego, CA). Comparisons among different groups were analyzed by either one‐way or two‐way ANOVA followed by Tukey's multiple‐comparison test. The level of significance was set at *P *<* *0.05 (two‐tailed).

## Results

**Figure 1:**
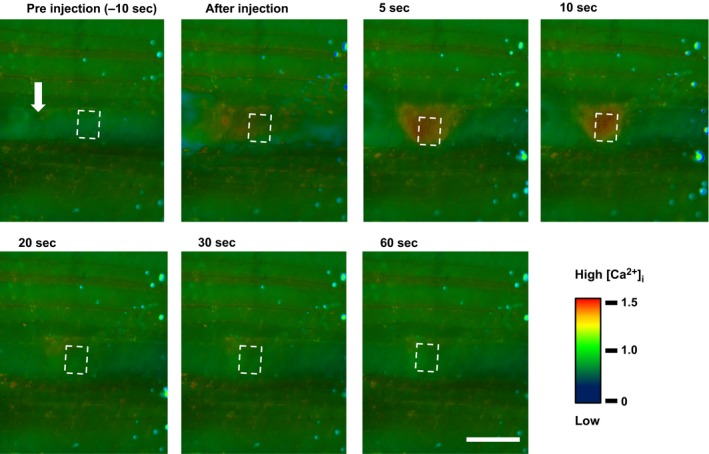
Representative example of changes in intracellular Ca^2+^ concentration ([Ca^2+^]_i_) induced by 2.0 mmol/L Ca^2+^ microinjection into a single rat spinotrapezius muscle myocyte. The injection site is shown with an arrow. The specific region of interest (width: 20 *μ*m, height: 80% of muscle fiber diameter) for the Ca^2+^ measurement was set to a distance of 40 *μ*m from the injection point. Scale bar = 50 *μ*m. Pseudocolor bar indicates the 340/380‐nm ratio value.

**Figure 2:**
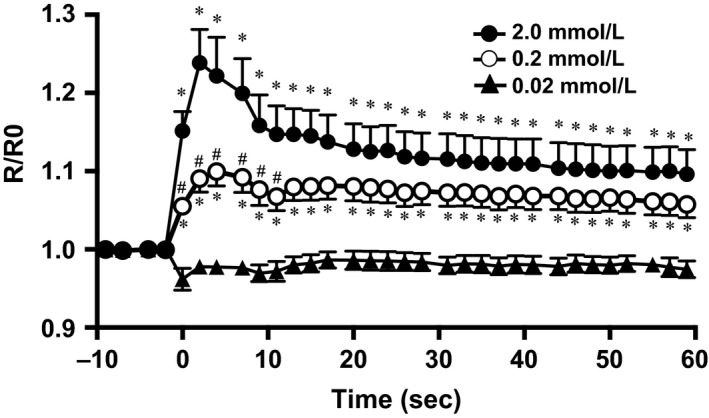
Influence of the different Ca^2+^ injections (0.02, 0.2 2.0 mmol/L) on intracellular Ca^2+^ concentration ([Ca^2+^]_i_) in vivo. Fluorescence was measured continuously during the 60 sec measurement interval and ratiometrically quantified data were graphed as changes from precondition (i.e. −10 sec) levels (R0). Values shown are means ± SE (*N* = 10, 0.02 mmol/L: *n* = 14, 0.2 mmol/L: *n* = 17, 2.0 mmol/L: *n* = 17). *N* = number of animals. *n* = number of muscle fibers measured. There was a significant interaction effect for treatment condition (0.02, 0.2 and 2.0 mmol/L) and time course of injection (*P* < 0.001). Significant difference between each conditions for the same time points: *P <* *0.05 (vs. 0.02 mmol/L), ^#^ *P *<* *0.05 (vs. 2.0 mmol/L).

In contrast to the Ca^2+^‐free and 0.02 mmol/L Ca^2+^ injections which caused no detectable [Ca^2+^]_i_ change, 0.2 and 2.0 mmol/L injections elicited significant increases in [Ca^2+^]_i_, indicated by 9.9 ± 1.8% and 23.8 ± 4.3% increases in R/R0 within 1–2 sec after injection, respectively (both *P *<* *0.0001, two‐way ANOVA, Figs. 1, 2). Interestingly, the [Ca^2+^]_i_ increase declined subsequently but [Ca^2+^]_i_ did not return to resting levels within the 60 sec observation period.

**Figure 3:**
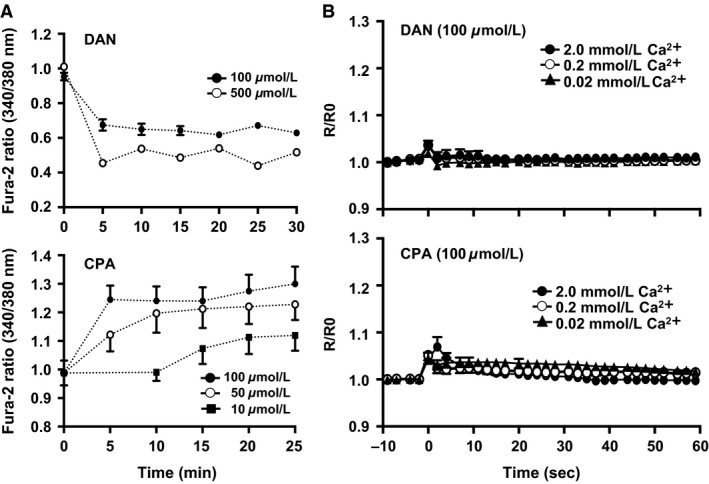
(A) The dose–response curves show the pharmacologic function of DAN (100, 500 *μ*mol/L) and CPA (10, 50, 100 *μ*mol/L) in the in vivo environment. (B) Influence of the DAN, top panel and CPA, bottom panel in vivo on intracellular Ca^2+^ concentration ([Ca^2+^]_i_). Values shown are means ± SE (DAN condition: *N* = 7, 0.02 mmol/L: *n* = 14, 0.2 mmol/L: *n* = 18, 2.0 mmol/L: *n* = 18, CPA condition: *N* = 7, 0.02 mmol/L: *n* = 15, 0.2 mmol/L: *n* = 15, 2.0 mmol/L: *n* = 15). *N* = number of animals. *n* = number of muscle fibers measured. CPA, cyclopiazonic acid; DAN, dantrolene sodium.

The functional concentrations of each inhibitor (DAN and CPA) were verified under in vivo conditions before Ca^2+^ injection (Fig. 3A). As evident from Figure 3B, the increment of [Ca^2+^]_i_ with Ca^2+^ injection was barely detectable after DAN treatment (2.0 mmol/L: 3.4 ± 1.2%, 0.2 mmol/L: 3.6 ± 0.8%, 0.02 mmol/L: 1.9 ± 0.7% vs. unblocked control). With CPA‐induced SERCA blockade, the [Ca^2+^]_i_ increase after injection was substantially blunted compared with the unblocked control condition (i.e., control 2.0 mmol/L Ca^2+^ injection, 23.8 ± 4.3% vs. CPA, 5.3 ± 1.0%, *P* < 0.05).

**Figure 4:**
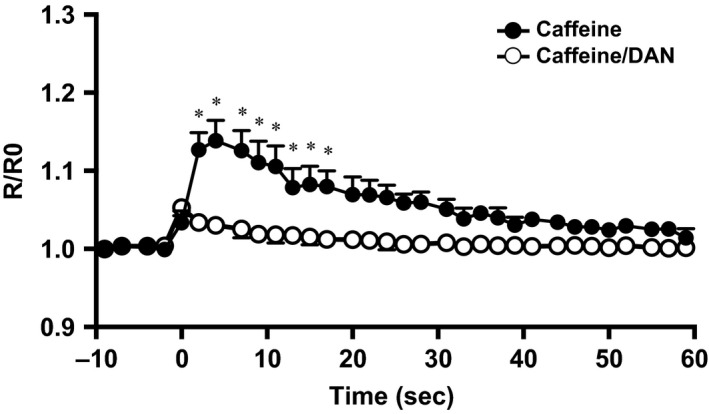
Influence of caffeine injection with/without DAN in vivo on intracellular Ca^2+^ concentration ([Ca^2+^]_i_). Values shown are means ± SE (caffeine condition: *N* = 5, *n* = 9, caffeine/DAN condition: *N* = 5, *n* = 8). *N* = number of animals. *n* = number of muscle fibers measured. There was a significant interaction effect for treatment condition (caffeine injection with/without DAN) and time course of injection (*P* < 0.001). Significant difference between each condition at the same time point: *P <* *0.05 (caffeine injection with vs. without DAN). DAN, dantrolene sodium.

Caffeine injection induced a 13.8 ± 2.6% [Ca^2+^]_i_ increase above baseline (Fig. 4). Unlike the 0.2 mmol/L and 2.0 mmol/L injectates, this [Ca^2+^]_i_ increase resolved to baseline within 60 sec (compare Figs. 2, 4). DAN blockade of RyR almost completely abolished the caffeine‐induced [Ca^2+^]_i_ increase (Fig. 4).

**Figure 5:**
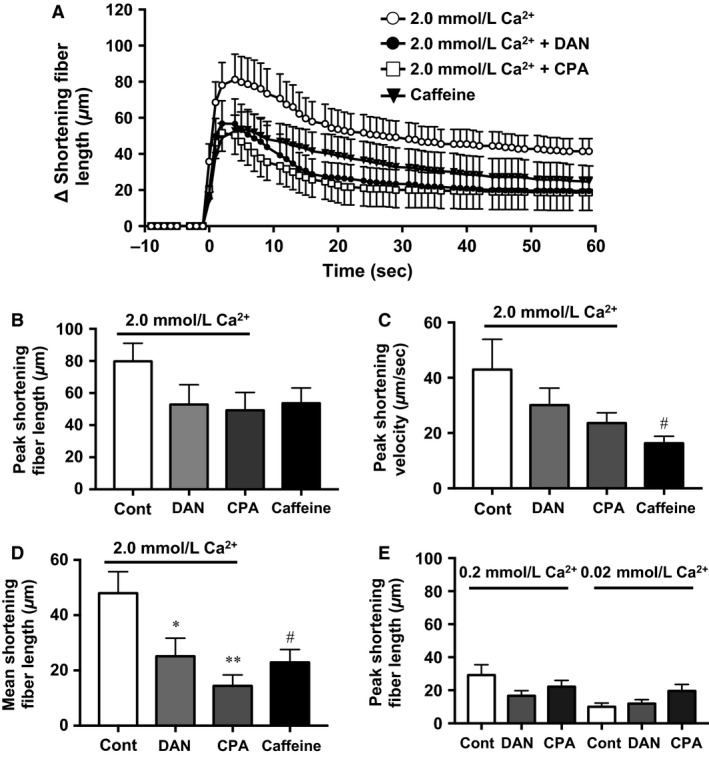
(A) Changes of muscle fiber length (i.e., extent of shortening) during each injection protocol (Ca^2+^, Ca^2+^+DAN, Ca^2+^+CPA, and caffeine). Values shown are means ± SE (Ca^2+^: *n* = 12, Ca^2+^+DAN:* n* = 14 Ca^2+^+CPA:* n* = 15, caffeine: *n* = 9). *n* = number of muscle fibers measured. B, C, and D: Shortening dynamics at 2.0 mmol/L Ca^2+^ and caffeine injection. (B) Bar graph shows peak shortening length. There was no statistically significant difference among conditions. (C) Bar graph shows peak shorting velocity. The Caffeine condition tended to be slower than 2.0 mmol/L Ca^2+^ control (^#^ *P* = 0.060). (D) Bar graph shows mean fiber length shortening during 60 sec following injection. Significance compared with Ca^2+^ condition, *P < 0.05, **P < 0.01. The Caffeine condition tended to be shorter than the Ca^2+^ condition (^#^ *P* = 0.059). (E) Bar graph shows peak shortening length for 0.2 mmol/L and 0.02 mmol/L Ca^2+^ conditions. Significant changes with the inhibitors were not found in either Ca^2+^ concentration conditions. CPA, cyclopiazonic acid; DAN, dantrolene sodium.

2.0 mmol/L Ca^2+^ injection induced a rapid (peak velocity, 43.0 ± 11.0 *μ*m/sec) and substantial (peak shortening length, 89.5 ± 15.1 *μ*m, mean shortening length 48.0 ± 7.7 *μ*m) fiber shortening as seen in Figure 5. Two‐way repeated‐measures analyses revealed a significant effect for DAN (*P *<* *0.03, vs. Ca^2+^ alone condition) and CPA (*P *<* *0.03, vs. Ca^2+^ alone condition) on shortening fiber length. Specifically, the mean shortening length was significantly reduced in both DAN (25.1 ± 6.6 *μ*m) and CPA (14.4 ± 4.0 *μ*m) compared with 2.0 mmol/L Ca^2+^ alone (Fig. 5D).

For the caffeine condition, compared with the 2.0 mmol/L Ca^2+^ injection, there was a strong tendency for a reduced shortening (22.9 ± 4.6 *μ*m, *P *=* *0.059, Fig. 5D) and a slower velocity of shortening (16.3 ± 2.5 *μ*m/sec, *P *=* *0.060, Fig. 5C).

## Discussion

This investigation represents the first in vivo imaging of [Ca^2+^]_i_ dynamics associated with direct single fiber Ca^2+^ loading in an in vivo skeletal muscle of rats. The principal original finding was that the [Ca^2+^]_i_ dynamics following Ca^2+^ injection reflect Ca^2+^ released by SOICR rather than the effects of the injected Ca^2+^ itself. This schema is supported by the almost complete abolition of [Ca^2+^]_i_ increase consequent to RyR and SERCA inhibition.

#### [Ca^2+^]_i_ response to Ca^2+^ injection

In the absence of intramyocyte (e.g., troponin) Ca^2+^ binding as well as SR release and sequestration processes the change in [Ca^2+^]_i_ should directly reflect the amount of injected Ca^2+^ within the cytoplasmic distribution volume. For instance, in a myocyte that has a 60 *μ*m diameter and length 700 *μ*m, the volume will be approximately 2.0 × 10^−3^ μL. When 0.78 × 10^−3^ *μ*L of 0.02, 0.2, and 2.0 mmol/L Ca^2+^ solution was injected, it is estimated that the [Ca^2+^]_i_ will increase to 0.0057, 0.057, and 0.57 mmol/L, respectively. As can be seen from Figures 2 and 3, however, there was no proportional relationship between injected [Ca^2+^] and the resultant rise in [Ca^2+^]_i_, providing evidence for the presence of a complex Ca^2+^ homeostasis system. That the 0.02 mmol/L injection did not elevate [Ca^2+^]_i_ indicates that either any [Ca^2+^]_i_ increase was below the sensitivity of our detection system or, alternatively, the injected Ca^2+^ was removed by Ca^2+^‐troponin binding and/or sequestration processes by SR and mitochondria. In contrast, both 0.2 mmol/L and 2.0 mmol/L injections invoked a rapid and substantial increase in [Ca^2+^]_i_ (Fig. 2) that was almost entirely prevented by either RyR (DAN) or SERCA (CPA) inhibition (Fig. 3). This observation provides strong evidence that a functional and robust SOICR is present in rat myocytes in vivo.

Ca^2+^ itself modulates RyR channel activity (Gehlert et al. 2015) being stimulatory at low Ca^2+^ (~1 *μ*mol/L) and inhibitory at extremely high Ca^2+^ (~1 mmol/L) (Meissner 1986). DAN inhibits skeletal muscle L‐type Ca^2+^ currents by disrupting communication between RyR1 and Ca(V)1 which provides the only treatment for malignant hyperthermia resulting from RyR1 mutations (Zhao et al. 2001; Bannister 2013). Although the precise molecular mechanism(s) for DAN's RyR1 functional action remain to be defined (Nelson et al. 1996; Oo et al. 2015), discrete findings within isolated muscle and skinned muscle fibers offer pertinent clues. For instance, in E–C coupling, DAN diminishes the twitch tension much more than the tetanic tension (Leslie and Part 1981) and both reduced temperatures (22°C) (Ohta et al. 1990; Krause et al. 2004; Kobayashi et al. 2005) and the presence of Mg^2+^ (Ohta et al. 1990; Owen et al. 1997) impair DAN's ability to inhibit RyR1. Unfortunately, as mentioned in the Introduction, in vitro conditions present in those investigations are expected, in and of themselves, to perturb [Ca^2+^]_i_ and RyR regulation. Specifically, RyR is a redox‐sensitive channel and myocyte redox regulation is dependent on O_2_ (and thus O_2_ partial pressure, PO_2_) (Salama et al. 2000; Powers et al. 2011). Although physiological microvascular PO_2_ are 20–30 Torr in the in vivo spinotrapezius muscle (Kano et al. 2005) and intramyocyte PO_2_ must be even lower, studies of isolated skeletal muscles are routinely performed in hyperoxic environments (95% O_2_, >600 Torr). This realization tempers confidence in translating ex vivo findings to the in vivo environment. Notwithstanding this consideration, the present investigation has extended previous observations regarding [Ca^2+^]_i_ control and represents the first investigation to provide evidence for SOICR function in vivo.

#### The effects of RyR and SERCA inhibition on the [Ca^2+^]_i_ response and contractile function

We hypothesized that SOICR function would be abolished when Ca^2+^ release from the SR is inhibited, either directly via the blockade of RyR (DAN), or indirectly via the depletion of Ca^2+^ from the SR (CPA). This hypothesis was supported by the almost complete abolition of [Ca^2+^]_i_ increases by DAN and CPA applied separately.

SOICR has been observed in RyR2 containing cardiomyocytes (Diaz et al. 1997; Eisner et al. 2009) and occurs consequent to RyR opening when the stored Ca^2+^ content exceeds some threshold level. SOICR has been observed for RYR1 in skeletal muscle myocytes as well as RyR3 in smooth muscle (Chen et al. 2014). As we added an additional Ca^2+^ load to the myocytes, we considered this to be the basis for triggering SOICR. Recently, Cully and colleague observed in rodent skinned fibers that a local [Ca^2+^]_i_ increase via SOICR was propagated by movement of Ca^2+^ inside the SR (Cully et al. 2014). A similar phenomenon was identified in rabbit ventricular myocytes where local SR Ca^2+^ uptake by SERCA facilitated propagation of cytosolic Ca^2+^ waves via luminal sensitization of the RyR (Maxwell and Blatter 2012). These cytoplasmic Ca^2+^ waves can be explained by discrete fluctuations of Ca^2+^ inside the SR network. In the present investigation, we have demonstrated that, following injection of 0.2 and 2.0 mmol/L Ca^2+^ solution, pharmacologic inhibition of SR Ca^2+^ release by DAN and uptake by CPA largely, but not totally, prevented subsequent [Ca^2+^]_i_ increase (Fig. 3, lower panel). As injected Ca^2+^ would be instantly sequestered by SERCA, when allowed to function normally (i.e., absence of CPA) this would acutely increase net SR Ca^2+^ stores. Consequently, local Ca^2+^ release following Ca^2+^ injection would be triggered by the SOICR system and contribute to the contractile behavior evident in Figure 5. Interestingly, even in the presence of DAN or CPA 40–60% of the control muscle contractile response (extent of shortening and shortening velocity) was preserved. The most likely explanation for this behavior is that the injected Ca^2+^ bound immediately to troponin and directly facilitated contraction.

#### Consideration of the experimental model

We examined Ca^2+^ release with caffeine to confirm the physiological response of the in vivo experimental model. Caffeine increases the sensitivity of RyR for Ca^2+^ (Rousseau et al. 1988; Zucchi and Ronca‐Testoni 1997; Murayama et al. 2000) and caffeine‐induced Ca^2+^ transients are evident in myotubes and adult skeletal muscle (Endo et al. 1970; Horiuti 1988; Lamb et al. 2001). In this study, under the in vivo control condition, there was a significant increase in [Ca^2+^]_i_ after caffeine injection, and this was markedly attenuated by DAN (Fig. 4). That Ca^2+^ release by caffeine could be inhibited by DAN provides additional evidence that the Ca^2+^ release via SR is present and functional in vivo. Also, the muscle contraction induced by caffeine injection is most likely the result of RyR‐released Ca^2+^. The peak shortening velocity and extent of fiber shortening with caffeine tended to be slower and reduced compared with the control Ca^2+^ condition (*P* = 0.06, Fig. 5 panel C) which might be associated with inertia in the Ca^2+^ release system resulting from caffeine‐induced RyR sensitization. Under these circumstances, it would be expected that the SOICR Ca^2+^ release rate would be far slower than VICR. However, resolution of the mechanistic basis for this behavior will require more precise temporal measurements of the contractile response than attempted, or possible, herein.

The [Ca^2+^]_i_ in mammalian myocyte is around 20–50 nmol/L under resting conditions, while the peak [Ca^2+^]_i_ increases to the low *μ*mol/L level during tetanic stimulation (Allen and Westerblad 2001). Computational modeling estimates that the total released SR Ca^2+^ is around 0.35 mmol/L during tetanic stimulation in intact fast‐twitch mouse myocytes (Baylor and Hollingworth 2003). It is quite possible therefore that the local injection of 2.0 mmol/L Ca^2+^ may be beyond the range seen physiologically. Although highly concentrated Ca^2+^ (~1 mmol/L) has been shown to inhibit rather than facilitate RyR function (Meissner 1986), the present results clearly demonstrate facilitation of RyR Ca^2+^ release. Therefore, even though we elected to inject 2.0 mmol/L Ca^2+^ the volume was extremely small (0.78 nL) and the resultant free [Ca^2+^]_i_ is expected to be reduced immediately by intramyocyte buffering mechanisms and dilution within the far larger cytosolic volume.

Mg^2+^, Zn^2+^, NO, and ATP are known modulators of the RyR function (Xia et al. 2000; Lanner et al. 2010; Suhr et al. 2013) and perturbations in the concentrations of these molecules, and therefore Ca^2+^ regulation, is inevitable when the in vivo myocyte environment is lost. For instance, the ATP requirement for the SR Ca^2+^ pumping, required to maintain Ca^2+^ homeostasis, demands up to 40–50% of resting cellular energy consumption (Smith et al. 2013). Even if it is possible to provide sufficient O_2_ in vitro to sustain this activity disruption of the intracellular PO_2_ (by hyperoxia and/or hypoxia) will invariably alter metabolic regulation, intramyocyte redox and the intramyocyte milieu (e.g., (Hogan et al. 1992; Wilson et al. 1977). This consideration demands careful interpretation of in vitro data when applying them to in vivo physiological mechanisms and is especially pertinent for understanding in vivo muscle Ca^2+^ homeostasis. A signatory advantage of the present investigation is having a preparation that maintains close‐to‐physiological intramyocyte homeostasis especially as regards ion balance and energy supply.

In conclusion, in the absence of VICR, we demonstrated, for the first time, evidence for SR SOICR in in vivo single mammalian skeletal muscle myocytes. The SOICR demonstrated induced elevated [Ca^2+^]_i_ and significant myocyte shortening; both of which were diminished substantially by RyR blockade or SERCA inhibition.

## Conflict of Interest

None declared.
